# Genotoxic assessment of a *Cannabis sativa* L. extract

**DOI:** 10.1080/13880209.2025.2499075

**Published:** 2025-05-06

**Authors:** Mark J. Tallon, Robert B. Child, Jason L. Blum

**Affiliations:** ^a^ Legal Products Group Ltd, Ely, UK; ^b^ School of Chemical Engineering, University of Birmingham, Birmingham, UK; ^c^ Product Safety Labs, Dayton, NJ, USA

**Keywords:** CBD, cannabidiol, toxicity, subchronic, safety, cannabis

## Abstract

**Context:**

As a naturally occurring terpenoid found in *Cannabis sativa* L. (Cannabaceae), cannabidiol (CBD) has gained public and industry interest for the purposes of personal well-being as a foodstuff and pharmaceutical. Despite a number of publications on CBD toxicology, many have significant limitations, especially those relating to genotoxicity. These include poor characterization of the CBD extract and/or lack rigor in conforming to accepted regulatory guidelines and best practice. A number of regulatory agencies have highlighted these issues and requested additional genotoxicity data to help ensure the safe use of CBD.

**Objective:**

To provide insights into the genotoxicity of a CBD isolate and its lipid carrier.

**Materials and methods:**

We have conducted an *in vitro* mammalian cell micronucleus (OECD 487) and a bacterial reverse mutagenicity assay (Ames test) (OECD 471) in a CBD isolate (97% > CBD) with its carrier.

**Results:**

The samples tested were non-mutagenic, as determined in the Ames test. The *in vitro* micronucleus assay conducted was negative for genotoxicity, with no statistically significant increases in the incidences of micronucleated cells observed at any dose compared to negative controls.

**Conclusions:**

These studies confirm that this CBD rich isolate in combination with its carrier, are unlikely to post any genotoxic hazard at exposure levels expected in foods.

## Introduction

The plant *Cannabis sativa* L. (Cannabaceae) contains a complex mixture of psychoactive and non-psychoactive chemical constituents. This dioecious species has a broad spectrum of phytochemicals in part due to its chemotype (Lehmann and Brenneisen 1995), and recent interest from the food and pharmaceutical industry has focused on the unique class of terpenophenolic compounds called cannabinoids. This class of compounds rose to government and scientific prominence following the discovery of tetrahydrocannabinol (Δ9-THC), which is the main psychoactive constituent resulting in the classification of cannabis as a narcotic (Brown 1998). Over 560 compounds, including over 100 cannabinoids, have been identified in Cannabis sativa (Morales et al. 2017). Of these, cannabidiol (CBD) has garnered the most attention due to its therapeutic approval as an epilepsy drug (Morano et al. 2020). Currently, there are attempts to obtain authorization for food use of CBD in the UK, Europe (Tallon 2020), and USA (Zawatsky et al. 2024), in line with approvals already permitted in Canada (Government of Canada n.d.), Australia (TGA and Australian Government Department of Health and Aged Care Therapeutic Goods Administration 2020), and Latin America (Ereño 2021).

Despite attempts to obtain food use approval for CBD in the EU and UK, it remains illegal under novel foods legislation (European Parliament, Council of the European Union 2015). Previous analysis of the available data by risk managers at EU level raises a number of concerns but does not address on a case-by-case basis the implications of different cannabis extracts and doses on toxicological profiles (EFSA Panel on Nutrition, Novel Foods and Food Allergens (NDA) et al. 2022). There are limited data in the public domain on the safety of CBD, but over the past 5 years, the understanding of CBD safety has improved greatly, although further investigations are necessary to gain insights into potential genotoxic effects.

Over the past decade CBD based food products have become extremely popular resulting in widespread and high frequency of use. For example, a survey of 45,300 adults from the United States and Canada (aged 16 and over) demonstrated that 16.2–26.1% had used CBD supplements in the previous 12 months (Goodman et al. 2022). Similarly, the Brightfield Group (2023) estimated 15% of Americans (49.8 million) used CBD regularly, with daily consumption levels between 20 and 1000 mg. Despite the popularity of CBD based food products, only seven studies have specifically assessed subchronic toxicity and/or the genotoxicity of orally administered hemp extracts (Marx et al. 2018; Dziwenka et al. 2020, 2021; Clewell et al. 2023; Henderson, Lefever, et al. 2023; Henderson, Welsh, et al. 2023; Tallon and Child 2023). The hemp extracts used in these investigations all had different compositions, due to differences in the plant part, plant species, and/or the extraction process. In addition, early research trials used different genotoxic tests (e.g., bacterial reverse mutation *vs. in vitro* mammalian cell micronucleus). This severely limits how existing CBD genotoxicity data can be generalized to the diverse range of CBD products that are commercially available.

This paper presents the findings from 2 studies conducted in accordance with (OECD) guidelines, as part of a broader program of work on CBD accumulation and hemp extract safety (Child and Tallon 2022; Tallon and Child 2023). Due to the relative paucity of data on CBD genotoxicity (Clewell et al. 2023; Henderson, Lefever, et al. 2023; Henderson, Welsh, et al. 2023), the present studies provide valuable data on the genotoxicity of a CBD isolate and it’s lipid carrier, helping to address some of the genotoxicity data gaps identified by the European Food Safety Authority (EFSA Panel on Nutrition, Novel Foods and Food Allergens (NDA) et al. 2022). As such, the present investigation provides some of the safety data needed to facilitate the safe consumption of CBD based foods.

## Materials and methods

### Test material

The proprietary CBD test substance comprised a high purity CBD isolate with its lipid carrier [medium chain triglyceride (MCT)]. The CBD isolate was a whole plant ethanol extract of *Cannabis sativa* L. (Cannabaceae) and CBD, accounts for >97% of the total cannabinoids present (Batch No. 103501B, cbdMD Inc (Charlotte, NC, USA). The CBD isolate was diluted to a concentration of 31–33% using MCT oil supplied by cbdMD Inc (Charlotte, NC, USA). The CBD concentration was independently determined by liquid chromatography–diode array detection (Vaclavik et al. 2019) against a CBD reference standard (Catalogue No. C6395), Sigma-Aldrich, Saint Louis, MI, USA) and a Certificate of Analysis issued (Eurofins, Charlotte, North Carolina). The CBD test substance was determined to be free from bacterial, yeast, metal, mycotoxins, and pesticide residue contamination (Eurofins, Charlotte, North Carolina). The stock CBD test substance was stored in ambient conditions in the dark and prepared on the day of experimentation.

Under conditions of commercial packaging, the CBD content of the CBD test substance was stable for more than 12 months, based on testing five representative batches. Three batches of the CBD test substance were utilized in the current investigation. Additional cannabinoids present in the isolate included cannabigerol (1.71%), cannabinol (0.47%), and cannabidivarin (0.22%). The isolate also contained other minor fatty acids, sterols, and terpenes.

### Statistical analyses

All statistical analyses were conducted with Provantis^®^ version 9 statistical software (Instem LSS, UK), with statistical significance set at *p* < 0.05. Unless otherwise stated, data are presented as means ± the standard deviation (*SD*), with details of the specific statistical tests performed in the methods section for each experiment.

### Genotoxicity studies

The genotoxicity of the CBD test substance was performed in accordance with OECD 471 (OECD 2020) and OECD 487 (OECD 2023). Similarly, all procedures were in compliance with the OECD principles on Good Laboratory Practices. They were also in line with Redbook 2000 (Food and Drug Administration 2000) and International Council for Harmonization (ICH) Harmonized Tripartrite Guideline S2 (R1) guidance on genotoxicity testing (ICH Harmonised Tripartite 2012). All physiochemical and biological analyses were carried out in ISO/IES 17025:2017 accredited facilities.

### In vitro *mammalian cell micronucleus test (OECD 487)*

The study was conducted to evaluate the genotoxicity (chromosomal damage) caused by the CBD test substance in an *in vivo* micronucleus assay (Kirsch-Volders et al. 2003; Bryce et al. 2007). The design was based on OECD guideline 487 (OECD 2023), using human lymphoblastoid cells (TK6 Cells, Cell line number CRL-8015) obtained from American Type Culture Collection (ATCC; Manassas, VA, USA).

An initial dose range finding study of 1–2000 μg/mL (30% CBD in MCT oil) was used to define the correct dosage for the *in vitro* micronucleus test. The metabolic activation system was provided by the addition of phenobarbital/benzoflavone-induced Sprague Dawley rat liver S9 (Moltox, Boone, NC, USA) mixed with appropriate cofactors (Regensys™ NADPH Regeneration System Reagents, Moltox, Boone, NC, USA) at a final concentration of 1% S9 in cultures tested with metabolic activation. Positive controls were Vinblastine sulfate (Catalogue No. V1377, Sigma-Aldrich, target dose levels 3.75 and 0.75 ng/mL) for the 4- and 24-h treatment without metabolic activation, and Cyclophosphamide monohydrate (Catalogue No. C0768, Sigma-Aldrich, target dose levels 3ug/mL) for 4 h with metabolic activation. The vehicle control was dimethyl sulfoxide (Catalogue No. D4540, Sigma-Aldrich).

The micronucleus test evaluated concentrations of CBD test substance at concentrations of 60, 40, 20, 15, 10, 8, 6, 4, 3, 2, 1, and 0.5 μg/mL for 4 h with metabolic activation (+S9) and 30, 25, 20, 15, 10, 8, 6, 4, 3, 2, 1, and 0.5 μg/mL for 4 h and 24 h without metabolic activation (−S9). Cytotoxicity was measured as the relative survival of cells from treated cultures compared to cells from vehicle control cultures. Cytotoxicity exceeding OECD guidance of 55 ± 5% was not processed or analyzed (see Table 1).

**Table 1.** Treatment conditions and CBD test substance concentrations processed for micronuclei in the *in vitro* micronucleus study.

Group	Treatment conditions and concentrations		
	∼4 h without metabolic activation (-S9 mix)	∼4 h with metabolic activation (+S9 mix)	∼24 h without metabolic activation (−S9 mix)
DMSO (%)	1	1	1
Vinblastine (ng/mL)	3.75	–	0.75
Cyclophosphamide (μg/mL)	–	3	–
CBD test substance (μg/mL)	0.5, 1, 2, 3, 4, 6, 8, 10, 15, 20, 25, 30	0.5, 1, 2, 3, 4, 6, 8, 10, 15, 20, 40, 60	0.5, 1, 2, 3, 4, 6, 8, 10, 15, 20, 25, 30

For the micronucleus assay, quadruplicate cultures of exponentially growing cells seeded at 2.0 ± 0.25 × 10^5^ cells/mL in 96 well plates were exposed to the CBD test substance for 4 ± 0.5 h ± S9 and for 24 ± 1 h − S9. DMSO (Catalogue No. D4540, Sigma-Aldrich), with positive control exposures were included on each test plate.

Following 4 h exposure, the media were removed, the cells washed once with phosphate buffered saline at pH 7.4, and then re-suspended in complete TK6 medium for the remaining culture period. For 24-h exposures –S9, cells remained in the same exposure media for the entire culture period. For 4-h exposures ± S9, cells were returned to the incubator for additional incubation to achieve 1.5 cell cycles in the DMSO control cultures.

At the end of the culture periods, the cells were analyzed for cytotoxicity and micronucleus induction by flow cytometry. The flow-cytometry-based high-content cytotoxicity and micronucleus assay was performed using the *In Vitro* MicroFlow™ kit (Litron Laboratories, Rochester, NY, USA). The data were collected using a validated Becton-Dickinson FACSCantoII™ flow cytometer (BD Biosciences, San Jose, CA, USA). Unless limited by cytotoxicity, 5000 (±500) cells from each sample were analyzed for the frequency of micronuclei. Micronuclei were identified using the combination of size (as measured by light scatter) and fluorescence (based on differential staining steps), which differentiates debris and necrotic and apoptotic cells from ‘healthy’ cells with true micronuclei.

The negative controls were defined by <2% micronucleated cells, and the test concentration of the positive control used for each test condition was required to induce a statistically significant increase in micronuclei. For CBD test substance exposures, a response was considered positive if at least 1 test dose exhibited a statistically significant increase compared to the concurrent vehicle control, the increase was dose-related when evaluated with an appropriate trend test, and the results were outside the laboratory’s historical control range. If none of these criteria were met, the response was considered negative.

Statistical analysis of the data was performed using the Statistical Analysis System, version 9.2 (SAS Institute, Cary, NC, USA). Data homogeneity and normality of the vehicle control data were assessed using Levene’s test and the Shapiro–Wilk test, respectively. Homogeneous, normally distributed data and data that were homogeneous and normally distributed following transformation were analyzed using one-way analysis of variance (ANOVA), and treatment groups were compared to the appropriate control group using Dunnett’s multiple comparison test. Dose-dependent changes were evaluated using a linear regression model. Statistical significance was assessed at *p* < 0.025. A one-tailed *t*-test was used to verify a positive response to the positive controls (*p* < 0.05).

### Bacterial reverse mutagenicity assay (Ames test) (OECD 471)

The ability of the CBD test substance (30% CBD in MCT oil) to cause mutations was assessed in Ames tests using a plate incorporation and preincubation method. Bacterial strains used in all experiments were *Salmonella typhimurium* histidine-auxotrophic strains TA98, TA100, TA1535, TA1537, and *Escherichia coli* WP2 uvrA (respective Catalogue numbers 71-098 L, 71-100 L, 71-1535 L, 71-1537 L, 72-188 L) purchased from Molecular Toxicology, Inc (USA).

Based on prior work in the laboratory including range finding and solubility testing, the solvent dimethyl sulfoxide (DMSO) was used as the control and for dissolving the CBD test substance. In addition, historical laboratory data were compatible with the survival of bacteria and S9.

All strains were studied in the presence and absence of a rat liver metabolic activation system prepared from the liver’s of male Sprague-Dawley rats induced with phenobarbital and 5,6-benzoflavone (i.e., S9 mixture). The test followed OECD guideline no. 471 for the testing of chemicals (OECD 2020), in addition to following previously established conditions and methods (Ames et al. 1975; Maron and Ames 1983; Venitt and Parry 1984). The following strain-specific positive controls for the experiments in the absence of S9 mix used to demonstrate the effectiveness of the test were: Daunomycin for TA98 (6 μg/plate), Sodium azide for TA100 and TA1535 (1 μg/plate), ICR 191 acridine for TA1537 (1 μg/plate), and Methyl methanesulfonate for *E. coli* WP2 (mix) (2.5 μg/plate). The positive control for all bacterial strains in the presence of S9 mix was 2-aminoanthracene.

CBD test substance suspensions were prepared in a culture media and incubated at 37 °C for ∼2–6 h before treatments and growth were evaluated by spectrophotometric optical density. The CBD test substance was formulated in triplicate and as a solution in DMSO (4.8, 15.2, 47.9, 1517.5, 4795, and 15175 µg/plate). The previously prepared cultures were added to one set of each strain and after pouring, plates were placed on a level surface until the agar gelled. They were then inverted and incubated at ∼37 °C until growth was adequate for enumeration (∼65 h).

A confirmatory test was employed using pre-incubation modification of the plate incorporation test (above). The test of control substances, bacteria suspension, and S9/substitution buffer were incubated under agitation for ∼30 min at 37 °C prior to mixing with the overlay agar and before pouring it onto the plates, as described in the pre-incubation test.

After incubation, the number of colonies per plate was counted in triplicate; either manually and/or with the aid of a plate counter (Colony plate reader, Model Colony-Doc-It, Analytik Jena, USA). The mean relevant colony counts for each strain treated with DMSO should lie close to or within the expected range, taking into account the laboratory historical range and/or published values (Mortelmans and Zeiger 2000; Gatehouse 2012). The positive controls should produce a substantial increase in revertant colony numbers with appropriate bacterial strain confirming known mutagenicity. The Mutation Factor (MF) was calculated by dividing the mean revertant colony count for the corresponding concurrent DMSO control group. The result was considered positive (biologically relevant) if the CBD test substance showed a substantial increase in revertant colony counts, i.e., response MF > 2 for strains with mean value(s) outside the laboratory historical control range. The above increase must be dose related and/or reproducible, i.e., increases must be obtained at more than one experimental point. If the criteria above were not met, then the result was considered negative for mutagenicity.

## Results

### In vitro *mammalian cell micronucleus test (OECD 487)*

Excessive cytotoxicity was observed at 25 and 30 μg/mL in the 4- and 9-h treatment without metabolic activation, therefore 15 μg/mL was considered the highest analyzable concentration for this condition. The lowest test concentration to induce the OECD recommended range was 15 μg/ml and this was considered as the highest analyzable concentration. In the 4 h with S9, CBD test substance exposures at 60 μg/mL exceeded OECD cytotoxicity guidance, so 40 μg/mL was considered to be the highest analyzable concentration for this condition.

Following treatment with CBD isolate, no statistically significant increases in the incidences of micronucleated cells were observed at any dose level compared to current negative control values. In addition, there was no dose–response relationship, and all incidences of micronucleated cells were within historical controls, with the exception of 40 µg/mL for 4 h + S9 (Table 2). The 40 µg/mL CBD exposure at (4 h with S9) was statistically significant compared to the concurrent control; however, this was below the OECD cytotoxicity limit. Under the test conditions, there was no indication of clastogenicity or aneugenicity as measured by micronucleus induction following exposure to human lymphocytes for 4 h with or without S9 or 24 h in the absence of S9.

**Table 2.** CBD test substance *in vitro* human lymphocyte micronucleus assay (OECD 487).

Treatment group	4-h Without metabolic activation (−S9 mix)		4-h With metabolic activation (+S9 mix)		24-h Without metabolic activation (−S9 mix)	
	Cytotoxicity (%)	Mean MN (%)	Cytotoxicity (%)	Mean MN (%)	Cytotoxicity (%)	Mean MN (%)
DMSO	7.1	1.29	3.8	1.04	5.2	1.06
CBD test substance (μg/mL)						
0.5	4.4	0.82^†^	3.9	0.91^†^	7.4	0.39^†^
1	5.6	0.97^†^	3.9	1.23^†^	7.4	0.77^†^
2	6.1	1.16^†^	3.9	1.17^†^	7.3	0.51^†^
3	5.4	1.10^†^	4.3	1.16^†^	7.7	0.67^†^
4	6.9	0.67^†^	4.2	0.84^†^	8.3	0.37^†^
6	6.4	1.09^†^	4.3	1.41^†^	8.8	0.50^†^
8	7.9	0.81^†^	4.8	0.85^†^	10.5	0.90^†^
10	9.1	0.70^†^	6.3	0.73^†^	13.1	0.73^†^
15	14.4	0.79^†^	7.3	0.85^†^	18.6	1.22^†^
20	14.9	0.69	9.4	0.69^†^	20.2	1.43
25	20.4	0.95	–	–	35.4	4.19
30	20.5	0.66	–	–	48.2	7.80
40	–	–	23.1	2.10^‡^	–	–
60	–	–	38.5	1.08	–	–
VIN (3.75 ng/mL)	24.8	14.91	–	–	–	–
VIN (0.75 ng/mL)	–	–	–	–	22.5	22.78*
CP (3.0 μg/mL)	–	–	11.7	5.75*	–	–

Cytotoxicity (%): apoptotic/necrotic cells; MN: micronucleated cells; DMSO: dimethyl sulfoxide; CP: cyclophosphamide monohydrate; VIN: vinblastine sulfate.

* Significant at *p* < 0.05 (Student’s *t*-test).

^†^ Not significant (ANOVA/Dunnett’s for comparison against vehicle control and linear regression modelling for trend test, significance at *p* < 0.025).

^‡^ Significant (ANOVA/Dunnett’s for comparison against vehicle control and linear regression modelling for trend test, significance at *p* < 0.025.

### Bacterial reverse mutagenicity assay (Ames test) (OECD 471)

No substantial increases in revertant colony numbers were observed in any of the five tester strains following treatment with CBD test substance at any concentration, in the presence or absence of metabolic activation (S9) (see Table 3). Precipitation was observed in all strains at >4795 µg/plate in both conditions, but this did not obscure the counts or mutagenic evaluation. Individual plate contamination, which did not affect counts or mutagenic evaluation, was observed sporadically for *S. typhimurium* TA98, TA100, and TA 1537. No signs of toxicity were seen for any strain at any dose levels. There were no concentration-related or substantial CBD test substance-related increases in the number of revertant colonies observed with strains TA1535, TA1537, TA98, TA100, or *E. coli* WP2 uvrA in both the absence and presence of S9 using either the plate incorporation or the pre-incubation method.

**Table 3.** CBD test substance Ames test using *Salmonella typhimurium* and *Escherichai coli*: mean number and revertant/plate.

Treatment group	Dose (μg/plate)	Bacterial strain									
		S. *typhimurium* TA98		S. *typhimurium* TA100		S. *typhimurium* TA1535		S. *typhimurium* TA1537		*E. coli* WP2 uvrA	
		−S9	+S9	−S9	+S9	−S9	+S9	−S9	+S9	−S9	+S9
DMSO	N/A	37 ± 3.5	41 ± 2.3	107 ± 22.1	114 ± 7.4	17 ± 4	18 ± 4.7	10 ± 1.5	10 ± 6.2	34 ± 2.0	42 ± 2.9
CBD test substance	4.795	41 ± 3.8	47 ± 11.9	111 ± 4.9	114 ± 9.1	21 ± 5	12 ± 2.9	13 ± 5.3	7 ± 2.5	37 ± 6.7	53 ± 12.4
	15.157	40 ± 6.7	42 ± 8.0	105 ± 16.6	122 ± 6.7	20 ± 2.6	13 ± 2.1	11 ± 2.1	10 ± 3.2	47 ± 6.8	45 ± 4.4
	47.95	37 ± 9.1	47 ± 4.6	116 ± 2.6	124 ± 7.9	17 ± 6.1	13 ± 5.0	12 ± 4.6	9 ± 2.3	49 ± 6.7	58 ± 3.6
	151.75	40 ± 2.6	51 ± 5.3	105 ± 5.9	103 ± 20.6	18 ± 4.5	17 ± 2.5	6 ± 3.2	10 ± 1	52 ± 2.1	67 ± 2.5
	479.5	34 ± 5.8	50 ± 9.2	116 ± 10	107 ± 15.8	20 ± 3.1	13 ± 4.9	8 ± 3.6	14 ± 3.6	49 ± 3.1	58 ± 8.0
	1517.5	35 ± 4.6	48 ± 4.9	104 ± 5.8	96 ± 25.2	18 ± 1.5	13 ± 4.6	7 ± 1.7	10 ± 2	60 ± 2.6	54 ± 1.2
	4795	30 ± 10.6	36 ± 1.7	67 ± 11	74 ± 14	20 ± 2.9	14 ± 2.5	8 ± 1.2	4 ± 2	50 ± 6.4	66 ± 2.6
	15175	28 ± 3.8	41 ± 5.5	54 ± 10.2	47 ± 11.9	13 ± 2.1	11 ± 4.4	3 ± 1.2	6 ± 3.2	57 ± 8.0	53 ± 2.5
Daunomycin	6	1329 ± 106.8	–	–	–	–	–	–	–	–	–
2-Aminoanthracene	10	–	2800 ± 487.6		2568 ± 181.1	–	208 ± 17.1	–	217 ± 12.1	–	169 ± 17.2
Sodium azide	1.5	–	–	564 ± 56.6	–	656 ± 34.7	–	–	–	–	–
Methyl methanesulfonate	2.5	–	–	–	–	–	–	–	–	553 ± 46.9	–
ICR 191 acridine	1	–	–	–	–	–	–	414 ± 24	–	–	–

DMSO: dimethyl sulfoxide.

Data are means ± *SD*.

The number of histidine or tryptophan revertants (mutations) for each dose and strain with and without metabolic activation did not exceed the values for spontaneous mutations (baseline) (see Table 3). In conclusion, under test conditions, the CBD test substance was considered non-mutagenic at concentrations of up to 1517.5 µg/plate on bacterial tester strains *S. typhimurium* TA98, TA100, TA1535, TA1537, and *E. coli* WP2 uvrA.

## Discussion

In 2019, the European Commission who have responsibility for food safety in Europe updated the novel foods catalogue to classify CBD as novel (European Commission 2023). The Foods Standards Agency (FSA) is responsible for food safety in the UK and in January 2019 they also concluded that CBD isolates were ‘novel foods’. This made them illegal for sale as foodstuffs without prior authorization (Food Standards Agency 2020). Despite not issuing any authorizations, the FSA has allowed CBD food supplements to remain on sale at controlled dosages. This resulted in CBD becoming freely available in a wide variety of food formats, such that CBD products have become mainstream throughout the UK. Despite this, there are few only a small number of studies on CBD toxicology using validated methods that meet the high-quality standards required by UK and EU food regulators. The present investigation provides valuable data on the genotoxicity of a hemp isolate with a high CBD content (>97%) and it’s associated MCT oil carrier. This data is useful to help establish the safe use of food products containing similar CBD rich hemp isolates.

There have been global efforts to reduce and replace animal testing and improve alternative *in vitro* tests; which in addition to being more scientifically valid may also be more time and cost efficient (Chrz et al. 2020). Genotoxicity is a critical toxicological endpoint, highly relevant for public health and environmental protection, including the safety of consumers (Chrz et al. 2020). Applying the principle of the 3Rs, defined as Replacement, Reduction, and Refinement of animal testing (Russell and Burch 1959), provides an ethical basis to use laboratory animals for research. The Council Directive 2010/63/EU on the protection of animals supports efforts to identify alternatives to animal testing (European Parliament, Council of the European Union 2010; Beken et al. 2016). A range of *in vitro* testing approaches have been developed that are relevant to human biological systems; often incorporating human-derived tissue cultures or cell lines (OECD 2017).

Several *in vitro* genotoxicity tests have been accepted for regulatory purposes, including the *in vitro* mammalian cell micronucleus test (OECD 487) and Ames tests using *S. typhimurium* and *E. coli* (OECD 471). It has been proposed that to classify a substance as non-genotoxic, a combination of appropriate *in vitro* technologies should be employed (Kerecman Myers et al. 2017). Individual *in vitro* methods exhibit specific advantages and limitations, and when used in combination may increase both the sensitivity of safety evaluation (Bhagat 2018).

Risk assessors, including the European Food Safety Authority (EFSA), have attempted to review the available evidence on CBD safety (EFSA Panel on Nutrition, Novel Foods and Food Allergens (NDA) et al. 2022); however, the approach used was flawed. A key issue is that their review included studies that failed to meet the quality criteria required for regulated product submissions. As the findings from these studies may reflect experimental limitations rather than genuine CBD toxicity, their pooling with more rigorous studies could result in incorrect inferences to be drawn. In addition, data assessed in the gap analysis were not based on test material with a consistent composition, and/or only limited information on their purity is provided and in some cases included undisclosed impurities (EMA 2019). More recent reports by the Advisory Committee on Novel Foods and Processes (ACNFP) and FSA have developed an Acceptable Daily Intake (ADI). Unfortunately, there is a lack of transparency regarding the scientific studies this is based on and if the studies included were subject to peer review (ACNFP 2023). The ADI suggested in the FSA report provides the basis for policy decisions that currently limit CBD intake to a maximum10mg/d. However, this ADI has been provided without due consideration to the specific characteristics and composition of each cannabis extract (Choudry and Haynes 2024) or is in conflict with existing peer-reviewed data (Henderson, Vincent, et al. 2023). The present investigation was conducted to help determine if the CBD test substance was genotoxic, complementing previous work (Henderson, Vincent, et al. 2023). Demonstration that a test substance is not genotoxic is an essential pre-requisite to establishing an ADI.

A select number of *in vitro* toxicological tests have been accepted for regulatory purposes, including the mammalian cell micronucleus test (OECD 487) and Ames tests using *S. typhimurium* and *E. coli* (OECD 471). No single *in vitro* test allows detection of the wide range of structural DNA changes, manifest as adverse effects associated either with genotoxicity or mutagenicity (Nesslany 2017). Therefore, combinations of at least 2 validated *in vitro* tests of sufficient sensitivity and specificity are currently recommended (Chrz et al. 2020). Test substances may be considered as showing no genotoxic potential if all *in vitro* endpoints used are clearly negative, while at least two *in vitro* endpoints showing positive results may predict genotoxic potential (European Food Safety Authority 2011).

In keeping with current recommendations, the present investigation used two assays to assess the genotoxicity of the CBD test substance. The tests performed were the Ames test (OECD 471) and the *in vitro* micronucleus assay (OECD 487). These measure different parameters relevant to genotoxicity; OECD 471 detecting base changes or frameshift mutations in the genome, while OECD 471 detects chromosomal damage. Both of these assays assess genotoxic effects in the absence and presence of activated liver cells (Tables 1 and 2); the latter condition helping to simulate *in vivo* metabolism of the CBD test substance. This approach allows the genotoxic responses of compounds endogenous to the CBD test substance to be separated from those arising from hepatic metabolism. As the lipid carrier (MCT) was well established as not being genotoxic (Traul et al. 2000), therefore it was not necessary to perform additional genotoxic tests on this component of the test substance.

Many of the compounds present in hemp extracts demonstrate poor solubility in water (Štern et al. 2024). Previous toxicological studies using compounds with limited water solubility reported different effects between assays, which were thought to arise from failing to consistently get the test compound to dissolve in water (Chrz et al. 2020). To overcome this problem, DMSO was added to the test media in the present investigations, thereby solubilizing both hydrophobic and hydrophilic compounds in the CBD test substance. In addition, to increase the validity of the assay for *in vivo* conditions, this also ensured the cells were consistently exposed to the correct concentration of CBD from the CBD test substance.

The Ames test (OECD 471) has the ability to detect mutations induced by the CBD test substance if it causes base changes or frameshift mutations in the genome of bacterial strains. The *in vitro* micronucleus study (OECD 487) is performed to determine if the CBD test substance caused genotoxic effects *via* damage to chromosomes.

In both studies, the CBD test substance did not result in any evidence of genotoxicity compared to relevant controls. In the Ames test, the CBD test substance did not produce an increase in the number of revertants in the presence or absence of metabolic activation. Similar data by Henderson, Welsh, et al. (2023), Clewell et al. (2023), and Štern et al. (2024) are consistent with these findings, these authors using extracts containing 99–101%, 84–87%, and a 99% CBD, respectively. Unpublished data on the CBD drug Epidiolex^®^ also showed an absence of genotoxic effects up to 5000 ug/plate (CDER 2018); a dose slightly less than the highest used in this study. Cannabis extracts with CBD purity ranging from 7 to 63% CBD also showed no evidence of genotoxicity (Marx et al. 2018; Dziwenka et al. 2020, 2021; Štern et al. 2024).

Results from the *in vitro* micronucleus assay also demonstrated no cytotoxicity as indicated by no induction of micronuclei in TK6 cells in both 4- and 24-h assays, with and without S9 metabolic activation. This is in contrast to results from Russo et al. (2019) where HepG2 cells were positive for induction of micronuclei, as was the case in the bone marrow of mice (Zimmerman and Raj 1980). Russo et al. (2019) observed induction of cell death even at low concentrations, which is contradictory to the cytotoxic results, as the authors had reported no change in cell viability up to 54 μM. Recent data from Štern et al. (2024) using a cytokinesis-block micronucleus (CBMN) assay that allows for the detection of micronuclei resulting from chromosome breakage, found no significant increase at concentrations of up to 5 μg/ml. A paper by Zimmerman and Raj (1980) did not disclose if the source of CBD was plant-based or synthetic; furthermore, they failed to consider effects on erythropoiesis, which have the potential to produce false positives. These contrasting findings may reflect the greater validity of TK relative to HepG2 for assessing cytotoxicity (Zhang et al. 1995; OECD 2023).

In studies using the same cell types as in the present study, CBD was negative for inducing micronuclei at 27 h without metabolic activation and with and without activation at 4 h (Henderson, Welsh, et al. 2023). Marx et al. (2018) also found no evidence of mutagenicity using their CBMN assay with Epidiolex doses equivalent to 2000 mg/kg/day. The available data from the Epidiolex *in vivo* study showed no micronuclei induction, but the data may be considered flawed as they provide no confirmatory testing for the presence of CBD in the target tissues (bone marrow) (EMA 2019). Štern et al. (2024) in their CBMN assay also showed no significant increase in micronuclei after 24 h of exposure to either CBD isolate (99% CBD) or extract (63% CBD), at concentrations up to 5 μg/ml.

In contrast to earlier studies suggesting chromosomal damage by CBD (Russo et al. 2019), the CBD test substance used in the present investigation was shown to be non-mutagenic in a bacterial test system. This was supported by micronucleus testing demonstrating no clastogenicity or aneugenicity in human lymphocytes following exposure to the CBD test substance. These findings are consistent with earlier data showing that pure (99%) CBD does not result in DNA damage (Aviello et al. 2012) and a hemp extract containing CBD was found to be non-mutagenic, non-clastogenic and non-genotoxic (Štern et al. 2024).

In summary, the potential genotoxicity of the CBD test substance was assessed using a mammalian cell micronucleus test and Ames test. These tests respectively assess chromosomal damage and base changes, or frameshift mutations in the genome. Application of these assays to the CBD test substance did not produce any evidence of genotoxic effects, findings that are consistent with other studies.

## Data Availability

The data have been disclosed to Food Standards Agency (FSA) and the European Food Safety Authority (EFSA) but are confidential.
